# Epidemiology of suicide among children and adolescents in Austria, 2001–2014

**DOI:** 10.1007/s00508-016-1092-8

**Published:** 2016-10-14

**Authors:** Zrinka Laido, Martin Voracek, Benedikt Till, Jakob Pietschnig, Brigitte Eisenwort, Kanita Dervic, Gernot Sonneck, Thomas Niederkrotenthaler

**Affiliations:** 1 Suicide Research Unit, Institute of Social Medicine, Center for Public Health, Medical University of Vienna, Kinderspitalgasse 15, 1090 Vienna, Austria; 2 Wiener Werkstaette for Suicide Research, Vienna, Austria; 3 Department of Basic Psychological Research and Research Methods, School of Psychology, University of Vienna, Vienna, Austria; 4 Department of Applied Psychology: Health, Development, Enhancement, and Intervention, School of Psychology, University of Vienna, Vienna, Austria; 5 Department of Child and Adolescent Medicine (day unit, pediatric psychosomatics), Medical University of Vienna, Vienna, Austria; 6 Department of Child and Adolescent Psychiatry, Medical University of Innsbruck, Innsbruck, Austria; 7 Crisis Intervention Center Vienna, Vienna, Austria

**Keywords:** Suicide, Epidemiology, Children and adolescents, Austria, Prevention

## Abstract

**Background:**

Previous epidemiological analyses indicated a decreasing trend of suicide rates for 10–19-year-olds in Austria for the period 1970–2001. However, data from the new millennium are missing. This epidemiological update reports on youth suicide in Austria, covering the period 2001–2014 in order to inform suicide preventive interventions targeting adolescents.

**Methods:**

The data on registered suicides among Austrian minors (10–19 years) and the population size were obtained from Statistics Austria. Chi-squared tests were used to analyze the associations between the suicide methods used and sex, as well as between suicide methods and Austrian federal states. Spearman correlations were calculated to assess time trends in the suicide rates. One-way ANOVA was used to investigate annual suicide rates of age groups 10–14, 15–19, and 10–19 years across the nine Austrian federal states.

**Results:**

The total average suicide rate for Austrian minors was 4.57 per 100,000. The male–female ratio was 3.5:1. The total youth suicide rate and male suicide rate significantly declined from 2001 to 2014, whereas there were no significant changes in female rates. More than one third of suicides among Austrian youth occurred through hanging, whereas jumping in front of a moving object was the second-most common suicide method. A spring peak was found, with most suicides occurring in April and May.

**Conclusion:**

Suicide rates among minors in Austria continue to decrease. The present findings help to inform the ongoing implementation of the National Austrian Suicide Prevention Plan (SUPRA).

## Introduction

Youth suicide is a considerable public health problem in many Western countries including Austria. Suicide accounts for 8.5 % of all deaths among young people (15–29 years of age) globally and ranks consistently among the leading causes of death in this age group [1–3]. In 2013, the suicide rate for the age group 15–19 among 28 investigated EU countries was 4.51 per 100,000 [4]. Previous research on suicide among Austrian children and adolescents, which covered the period from 1970 to 2001 [5–8], reported a significant decrease in suicides among this population. An average total suicide rate of 6.2/100,000 was reported for Viennese minors aged 10–19 [6] and for Austrian children up to 14 years of 1.4 per 100,000 [7]. The most common suicide method among Viennese youth aged 19 and under for the period studied was jumping from a height [6], and for Austrian children up to 14 years of age it was hanging [7]. These epidemiological reports on youth suicide in Austria also showed that suicides were more common among boys than girls with a male–female ratio of approximately 2–3:1 [6, 7]. Furthermore, an increased frequency of suicides during school examination periods for Viennese youth aged 19 and under was observed [6], as well as spring and autumn peaks for Austrian children up to 14 years of age [7]. Whereas no information on the distribution of suicides among children and adolescents up to 19 years of age in Austrian federal states is available yet, suicides among children up to 14 were reported to be most common in Carinthia for the study period 1970–2001 [7]. An epidemiological update on youth suicide for Austria covering the ensuing years of the new millennium is needed, given that the last observation period was in 2002. This is important for clinical care, practitioners, public health measures, suicide prevention, and policy making in Austria.

The aims of the study were to assess: 1) the most recent suicide rates among Austrian children and adolescents (10–19 years), as well as gender and age (children vs. adolescents) differences; 2) the most common suicide methods used and possible recent changes; 3) possible regional differences in youth suicide rates between the nine Austrian federal states; and 4) the monthly distribution of suicides among Austrian minors**.**

## Methods

### Sample and procedure

Completed suicides among children and adolescents aged 10–19 years in Austria from 2001 to 2014 were investigated. The data on registered suicides among minors in Austria (10–19 years) and the size of the respective population groups were obtained from Statistics Austria. Suicide rates per 100,000 individuals were calculated for the age groups 10–14 and 15–19 years, as well as for these age brackets combined (10–19 years), separately for girls and boys, and furthermore separately for the nine higher-level administrative regions comprising Austria. Suicide rates were calculated as the annual number of suicide cases per 100,000 individuals of the specific reference population.

Suicide mortality as a cause of death was classified according to the International Classification of Diseases, Ninth Revision (ICD-9) for the year 2001. Therein, suicides are categorized under the codes E950–E959. For the period from 2002 on, the ICD-10 classification was used, with the codes X60–X84.

Reliability and validity aspects of the procedural details of suicide registration in Austria have been described in prior related research [7, 9, 10]. The gist of these findings shows that, in comparison with other countries, the reliability, sensitivity, and specificity of suicide certification in Austria is satisfactory [11, 12]. Of particular importance, in spite of a decreasing trend in the prevalence of autopsies [10], which parallels the temporal trend seen in autopsy rates in other (European as well as non-European) countries, Austrian autopsy rates are still comparatively high. According to the Austrian Health Care Acts, an autopsy by forensic pathologists is required if an obvious disease that should be the prime candidate as the underlying cause of death is not known. Consent from the relatives of the deceased individual is not necessarily required for this prescribed medicolegal procedure [13].

### Statistical analysis

One-way analyses of variance (ANOVA) were calculated to compare the annual suicide rates of the nine Austrian federal states. This was done for each of the three age groups (10–14, 15–19, and 10–19 years).

In order to assess time trends in the suicide rates, we calculated nonparametric correlation coefficients (Spearman’s rho) between yearly total, male, or female suicide rates and year of death, respectively.

Chi-squared tests were used to assess the associations between the suicide methods used and sex, as well as between suicide methods and Austrian federal states. In the case of too small cell numbers (i. e., expected values <5), we used Fisher’s exact test instead.

One-sample Kolmogorov–Smirnov tests for uniform distribution were used to test for evenness (vs. unevenness) in the monthly distribution of suicides. We performed all statistical analyses with SPSS, version 22.

## Results

During the period 2001–2014, 608 suicide cases among children and adolescents aged 10–19 years were officially registered in Austria. The breakdown by sex was 474 boys (78 %) vs. 134 girls (22 %). Of these suicides, 47 (7.7 %) were aged 14 and under, and among them were 30 (63.8 %) boys and 17 (36.2 %) girls. Further, 561 (92.3 %) suicides were aged 15–19, and of those there were 444 (79.1 %) boys and 117 (20.9 %) girls. There was no registered suicide case for children under 10 years of age during this period in Austria.

**Fig. 1:**
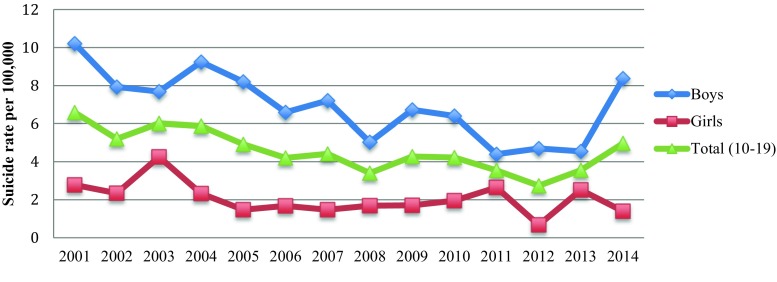
Suicide rates per 100,000 of Austrian minors during 2001–2014

Yearly suicide rates per 100,000 of Austrian children and adolescents in the age group 10–19 years, from 2001 to 2014, are shown in Fig. 1. For this period, the annualized average suicide rate for Austrian minors was 4.57 per 100,000. Sex-specific rates amounted to 6.96 per 100,000 for boys and 2.06 per 100,000 for girls. The male-to-female ratio was 3.5:1.

Both the yearly suicide rate for the total population of minors *(r*_s _(14) = −0.69, *p* = 0.006) and the boy-specific suicide rate (*r*_s_ (14) = −0.62, *p* = 0.02) decreased significantly over the 14-year period. Although the negative correlation between yearly suicide rate and year was seen for girls as well, this time trend was weaker and nominally not significant (*r*_s_ (14) = −0.43, *p* = 0.13).

Regarding age differences, for children under 14 years of age (*n* = 47), the calculated average suicide rate was 0.72 per 100,000 (male suicide rate 0.9, female suicide rate 0.5 per 100,000), while for adolescents (*n* = 561) the suicide rate was 8.20 per 100,000 (male suicide rate 12.7, female suicide rate 3.5 per 100,000).

With regard to suicide methods used in the youngest age group of 10–14 years, of the 47 registered suicides, 57.4 % (*n* = 27) were due to hanging, whereas 17.0 % (*n* = 8) were due to jumping from a height, 6.4 % (*n* = 3) were due to firearms, 2.1 % (*n* = 1) were due to poisoning, and 17.0 % (*n* = 8) were due to other methods.

### Suicide prevalence across Austrian federal states

The average suicide rates per 100,000 in children and adolescents for all nine Austrian federal states were calculated. The average total suicide rates (*F *(8, 117) = 3.14, *p* = 0.003) and the average suicide rates for 15–19-year-olds (*F* (8, 117) = 2.68, *p* = 0.01) differed significantly between regions.

**Fig. 2:**
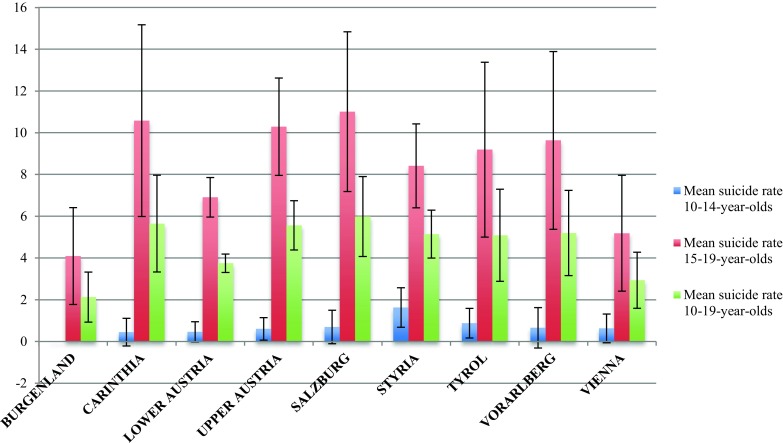
Mean suicide rates of Austrian minors by regions from 2001 to 2014, 95 % CI

**Fig. 3:**
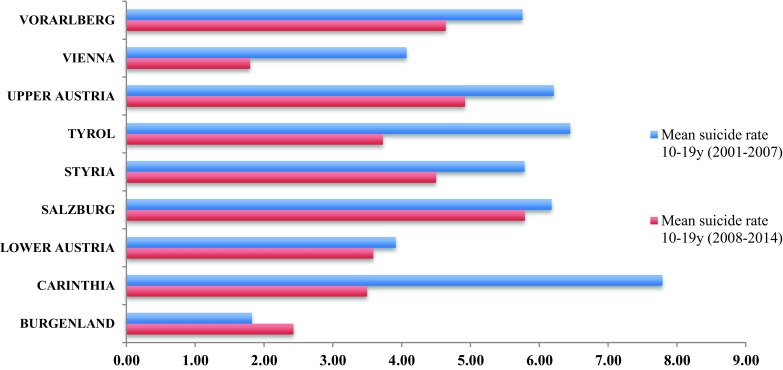
Mean suicide rates of 10–19-year-olds in Austrian federal states during two 7‑year periods

The lowest total suicide rates were observed for Burgenland (2.11) and Vienna (2.92). The highest total suicide rates were found in Salzburg (5.99) and Carinthia (5.72), although other regions had quite similar suicide rates, such as Upper Austria (5.58), Styria (5.19), Vorarlberg (5.19), and Tyrol (5.14) (Fig. 2). As indicated by the 95 % confidence intervals, particularly Salzburg, Styria, and Upper Austria had higher rates for minors compared with the other federal states (Fig. 2). As depicted in Fig. 3, the mean suicide rates of all federal states except Burgenland were lower in the second half of the observation period (2008–2014) than in the first half (2001–2007).

### Differences in suicide methods

The most frequently used suicide method among Austrian children and adolescents for both boys and girls was hanging, making up over a third of cases, followed by jumping and lying before a moving object (approximately every fifth suicide), and jumping from a great height (Table 1).

**Table 1:** Suicide methods in Austrian minors, total and by sex, 2001–2014

Suicide method	Boys [%] (474)	Girls (134)	Total (608)
Hanging	38.8 (184)	33.6 (45)	37.7 (229)
Jumping or lying/moving object	21.7 (103)	25.4 (34)	22.5 (137)
Jumping from a height	13.9 (66)	23.1 (31)	15.9 (97)
Shooting	13.1 (62)	2.2 (3)	10.7 (65)
Poisoning	2.9 (14)	9.7 (13)	4.4 (27)
Drowning	4.2 (20)	2.2 (3)	3.8 (23)
Other	3.6 (17)	2.2 (3)	3.3 (20)
Domestic and other gas	1.3 (6)	1.5 (2)	1.3 (8)
Cutting	0.4 (2)	0.0 (0)	0.3 (2)

The suicide methods used varied significantly between regions (χ^2^ (6, *N* = 608) = 30.08, *p* < 0.001). Similarly, in most Austrian federal states, the prevalent suicide method was hanging, followed by jumping and lying before a moving object, and jumping from a great height. A regional difference was apparent for Vorarlberg, where the most prevalent method of suicide was jumping from a great height, and in Vienna where the same method ranked second. In addition, in Lower Austria and Styria, the third most common method among minors was shooting (Table 2).

**Table 2:** Suicide methods in different regions of Austria (*N* of suicides), 2001–2014

Suicide method	Burgenland [%] (9)	Carinthia [%] (52)	Lower Austria [%] (98)	Upper Austria [%] (136)	Salzburg [%] (53)	Styria [%] (98)	Tyrol [%] (61)	Vorarlberg [%] (34)	Vienna [%] (67)
Hanging	33.3 (3)	30.8 (16)	39.8 (39)	45.6 (62)	37.7 (20)	33.7 (33)	49.2 (30)	14.7 (5)	31.3 (21)
Jumping or lying/moving object	11.1 (1)	28.8 (15)	25.5 (25)	19.8 (27)	32.1 (17)	26.5 (26)	14.7 (9)	26.5 (9)	11.9 (8)
Jumping from a height	0.0 (0)	25 (13)	12.2 (12)	11 (15)	11.3 (6)	11.2 (11)	13.1 (8)	41.2 (14)	26.9 (18)
Shooting	22.2 (2)	5.8 (3)	14.3 (14)	10.3 (14)	9.4 (5)	17.3 (17)	8.2 (5)	2.9 (1)	5.9 (4)
Poisoning	11.1 (1)	1.9 (1)	3.1 (3)	4.4 (6)	1.9 (1)	3.1 (3)	3.3 (2)	5.9 (2)	11.9 (8)
Other methods	22.2 (2)	7.7 (4)	5.1 (5)	8.8 (12)	7.5 (4)	8.2 (8)	11.5 (7)	8.8 (3)	11.9 (8)

### Monthly distribution of suicides

**Fig. 4:**
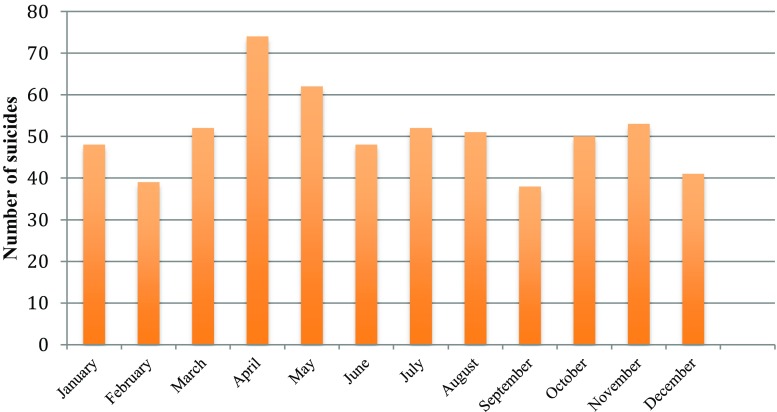
Monthly distribution of suicides

The monthly distribution of suicide cases differed significantly from a uniform distribution (*Z* = 1.44, *p* = 0.03). The highest numbers of suicides were observed for April (74 cases, 12.2 %) and May (62 cases, 10.2 %), followed by a decrease during the summer months until September. February, September, and December had the lowest number of registered suicides (Fig. 4).

## Discussion

Suicide rates among 10–19-year-olds in Austria decreased between 2001 and 2014. This observed trend is a continuation of a previously observed decrease in the respective suicide prevalence between 1970 and 2001 [7, 14] and, more generally, corresponds to a similar trend observed for the Austrian general population from 1986 to 2010 [5, 7, 8, 15]. The average total suicide rate for Austrian youth aged up to 19 of 4.5/100,000 in our study corresponds with the reported average youth suicide rate of 4.5/100,000 for EU countries [4]. In comparison to other OECD countries, the Austrian suicide rate for teenagers (15–19 years) has also been consistent with the average rate [16]. In several neighboring countries including but not limited to Germany, Switzerland, and Italy, the suicide rates of 15–19-year-old teenagers in the year 2010 were reported as lower than in 2000 [16, 17], indicating that the present decreasing trend has not been limited to Austria. The general decrease may be consistent with the implementation of national suicide prevention plans in many countries in recent years [3]. Further analyses are warranted to assess which countries have specifically implemented national or regional programs, and how these are related to suicide rates.

This epidemiological analysis offers important updated evidence to inform targeted interventions and encourages further suicide prevention initiatives. With hanging being the predominant suicide method among minors in Austria, and given that suicide means for hanging are ubiquitous [14], an early recognition and treatment of suicidal youths is of paramount importance. Psychological autopsy studies reveal that more than 90 % of youth suicides are associated with co-existing mental disorders [14, 18, 19]. Mental health problems become more prevalent during adolescence and in combination with psychosocial risk factors exacerbate vulnerability for suicidal behavior in adolescents. Psychosocial vulnerability includes depression, substance abuse, anxiety, aggression/impulsivity traits, family and school-related problems, and traumatic life events [18–23]. Moreover, several studies suggest that the early detection of parental risk factors and early intervention in high-risk families effectively reduces the burden of subsequent youth suicide [20–22]. In the context of health-care factors, the current precarious situation with an under-provision of ambulatory and specialist care from child and adolescent psychiatrists and psychotherapists in Austria has been frequently brought up in the public media in recent years [24, 25] and warrants urgent attention from political bodies, also in the context of the currently ongoing implementation of the Austrian National Suicide Prevention Plan.

In this observation period, particularly Styria, Salzburg, and Upper Austria seemed to have slightly higher child and adolescent suicide rates than the other federal states. While areas for improvement in psychiatric care and psychosocial services for children and adolescents have been identified for most federal states [25], the under-provision of medical specialists in child and adolescent psychiatry has been described as particularly critical for Styria, Salzburg, Vienna, and Burgenland [24]. However, particularly for Vienna and also for Burgenland, important improvements in some ambulatory services and other psychosocial services to adolescents have also been noted [25]. Interestingly, Vienna’s and Burgenland’s suicide rates were among the lowest in the observation period, which is consistent with findings from the adult population. Further analyses are warranted to scrutinize regional differences in child and adolescent suicide rates and their associations with population-based and health-care-related indicators. These considerations should also include small-area differences in urbanity [26] and considerations on biological differences within the population. In this context, Voracek et al. [27] analyzed differences in district-level standardized suicide rates during 1988–1994 across the whole population between the five major surname regions identified for Austria. Accordingly, Vienna, Burgenland, and the north of Lower Austria were observed as one region, and the mean suicide rate was lower than in other surname regions.

Our finding that jumping/lying before moving objects was the second most common suicide method among Austrian youths between 2001 and 2014 needs to be specifically addressed in suicide prevention initiatives. Research [28–31] has shown that restricting access to hotspots (i. e., installation of barriers) is effective in diminishing suicides by jumping. Other interventions like increasing the likelihood of helpful interventions by bystanders and providing guidance on responsible media reporting are also promising. Media reporting about suicides by jumping has been identified as a core risk factor for subsequent increases in suicides [32], and outreach media using media recommendations for the reporting on suicide has been shown to reduce subway suicides in Vienna [33, 34], where this suicide method ranks lower compared with the present national data. Although Eisenwort et al. [35] did not identify an association between suicide reports in German-speaking youth journals and subsequent youth suicides, adolescent age has been repeatedly described as a life period that is most susceptible to suicide contagion through media and other social networks [36].

In the previous epidemiological update of Dervic and colleagues regarding suicide in Austrian children aged 14 and younger [7], a concerning increase in the proportion of suicides by firearms was noted, from 9.3 % in the decade 1980–1989 to 20.6 % in the decade 1990–2001. This finding did not persist in the new millennium, where firearm suicides made up only 6.4 % of the suicides of children up to 14 years of age. However, hanging now makes up an even higher proportion of suicides in this youngest age group, 57.4 %, up from the previously reported 45.6 % in the period 1990–2001. The reduction in the proportion of firearm suicides in children may further support the effectiveness of the more stringent firearm legislation, which was implemented in Austria in 1997 [9, 37], and has resulted in a decrease in registered firearm licenses, firearm suicides, and homicides [37] and has also been shown to be associated with a mid-term reduction of youth suicides [9]. Because the availability of suicide means has an important and facilitating role in impulsive suicide among youngsters [6, 7, 38], further measures to increase safety with means and specifically reduce the availability of and access of firearms seem warranted.

Our finding that the majority of youth suicides occurred in April and May is in line with well-known reports on spring and autumn suicide peaks in the general population [5].

School interventions aiming to raise mental health and improve coping skills in all pupils through active peer intervention have been found to be effective in reducing suicide attempts and severe suicidal ideation in a large multicenter study in ten European countries. This study analyzed the impact of three types of interventions – i. e., peer interventions, a gatekeeper training module targeting teachers and other school personnel, and screening by professionals with referral of at-risk pupils – and found that peer intervention but not the other tested strategies were significantly related to a reduction in the aforementioned outcomes [39]. This study suggests that particularly peer interventions need to be strengthened in order to prevent suicide in school settings. However, the early detection of suicidal youth and the timely referral to treatment of psychiatric disorders by gatekeepers remain important aspects of prevention in young people [14, 25]. In this context, accurate training of community gatekeepers such as teachers, youth workers, school doctors, pediatricians, or general practitioners about suicidal behavior in adolescence and its association with mental health problems, environmental risk factors, and family and school problems, as well as how to provide proper management and referral, is necessary [14].

### Strengths and limitations

The representative nature of the data, which includes the whole population of interest, is a strength of the present study. Limitations include that data on relevant variables, such as the socioeconomic status of adolescents and their families, or the circumstances of suicide, were not available.

## Conclusion

This study provides an overdue update on the epidemiology of child and adolescent suicide in Austria, covering the period 2001–2014. Suicide rates among minors in Austria continue to decrease. The findings of the study can serve as a basis to inform the currently ongoing implementation of the Austrian Suicide Prevention Plan SUPRA, which lists children and adolescents as a specific target population [40].
